# Association of transcription factor *WRKY56* gene from *Populus simonii* × *P. nigra* with salt tolerance in *Arabidopsis thaliana*

**DOI:** 10.7717/peerj.7291

**Published:** 2019-07-09

**Authors:** Lei Wang, Wenjing Yao, Yao Sun, Jiying Wang, Tingbo Jiang

**Affiliations:** 1 Department of Biotechnology, Institute of Advanced Technology, Heilongjiang Academy of Sciences, Harbin, PR China; 2 State Key Laboratory of Tree Genetics and Breeding, College of Forestry, Northeast Forestry University, Harbin, PR China; 3 Bamboo Research Institute, Nanjing Forestry University, Nanjing, PR China

**Keywords:** *Arabidopsis thaliana*, *Populus simonii* × *P. nigra*, WRKY transcription factor, Salt stress

## Abstract

The WRKY transcription factor family is one of the largest groups of transcription factor in plants, playing important roles in growth, development, and biotic and abiotic stress responses. Many *WRKY* genes have been cloned from a variety of plant species and their functions have been analyzed. However, the studies on WRKY transcription factors in tree species under abiotic stress are still not well characterized. To understand the effects of the *WRKY* gene in response to abiotic stress, mRNA abundances of 102 *WRKY* genes in *Populus simonii* × *P. nigra* were identified by RNA sequencing under normal and salt stress conditions. The expression of 23 *WRKY* genes varied remarkably, in a tissue-specific manner, under salt stress. Since the *WRKY56* was one of the genes significantly induced by NaCl treatment, its cDNA fragment containing an open reading frame from *P. simonii* × *P. nigra* was then cloned and transferred into *Arabidopsis* using the floral dip method. Under salt stress, the transgenic *Arabidopsis* over-expressed the *WRKY56* gene, showing an increase in fresh weight, germination rate, proline content, and peroxidase and superoxide dismutase activity, when compared with the wild type. In contrast, transgenic *Arabidopsis* displayed a decrease in malondialdehyde content under salt stress. Overall, these results indicated that the *WRKY56* gene played an important role in regulating salt tolerance in transgenic *Arabidopsis*.

## Introduction

Plants are easily affected by biotic and abiotic stresses throughout their life cycle when in a natural environment. They have developed complex mechanisms that allow them to better adapt to the stresses. In various adaptive mechanisms, the transcription factor is a key regulatory component in response to stresses such as drought, salinity, cold, hormones, and pathogens. Transcription factors can specifically bind to cis-elements in promoters in order to regulate the expression of downstream target genes (Schwechheimer & Bevan, 1998). Since Paz-Ares first reported the transcription factor of maize in 1987 (Paz-Ares et al., 1987), a variety of transcription factor genes, such as those from the AP2/ERF (Li et al., 2015), WRKY (Zou et al., 2016), MYB (Dubos et al., 2010), and NAC families (Fang et al., 2008) have been isolated from plants. The WRKY is one of the largest transcription factor families in plants. The WRKY proteins are composed of a DNA-binding domain, a transcription regulation domain, a nuclear localization signal domain, and oligomerization sites or phosphorylation modification sites (Li & Zhou, 2014). The WRKY proteins are characteristic of one or two WRKY domains (the WRKYQK sequence) at the N-terminal, and a zinc finger motif (CX_4-5_CX_22-23_ HX_1_H type or CX_7_CX_23_HX_1_C) at the C-terminal. According to the number of WRKY domains and the features of the zinc finger motif, the WRKY transcription factors in plants can be divided into three groups: I, II, and III. Group I is composed of two WRKY domains and a zinc finger motif (C-X_4-5_-C-X_22-23_-H-X_1_-H). Group II contains one WRKY domain and a zinc finger motif which is same as group I. Group III includes one WRKY domain and a zinc finger motif (C-X_7_-C-X_23_-H-X_1_-C) (Chen et al., 2018).

The WRKY transcription factors can play either a positive or negative role (Dai et al., 2018; Bao et al., 2018) in response to stress. Some *WRKY* genes can be induced by environmental stresses and subsequently take part in the regulation of plant growth and development (Guo et al., 2015), defense responses to pathogens (Sarris et al., 2015), and can respond to abiotic stresses (Chen et al., 2015). During the stress response process, the WRKY transcription factors can positively regulate and initiate the plant defense responses through salicylic acid (Karim et al., 2015), jasmonate acid (Jiang et al., 2014), and ethylene signal transduction pathways (Chen et al., 2013). Conversely, many other WRKY transcription factors play negative roles in the transcriptional regulation of resistant genes. For example, CRK5, a cysteine-rich receptor-like protein kinase in *Arabidopsis* that is involved in ABA signaling, is regulated negatively by AtWRKY18, AtWRKY40, and AtWRKY60, collectively (Lu et al., 2016). Although many *WRKYs* have been cloned and characterized in plants, their regulatory roles are still poorly understood.

In an earlier work, the *WRKY56* in *Populus simonii* × *P. nigra* was found to be responsive to salt stress and the expression of the *WRKY56* was increased under salt stress by cDNA-AFLP and quantitative real-time reverse transcription polymerase chain reaction (qRT-PCR) (Wang et al., 2011, 2014). However, the function of *WRKY56* in salt tolerance was not clear at the time. In this study, different expression genes (DEGs) of *WRKY* were assayed by RNA-Seq to verify our primary work, and the *WRKY56* gene, a salt-induced gene, was cloned from *P. simonii* × *P. nigra* via RT-PCR. The expression of the *WRKY56* gene in leaves at different points of time under salt stress was also determined by qRT-PCR. The *WRKY56* gene was transferred to *Arabidopsis thaliana* by the floral dip method. In order to shed light on the function of *WRKY56*, the growth and physiological traits of transgenic *Arabidopsis* were investigated. This study will provide experimental evidence for better understanding the biological function of the *WRKY56* gene as a valuable genetic resource for plant breeding applications.

## Materials and Methods

### Plant growth and stress treatment

The twigs of *P. simonii* × *P. nigra* from the same clone were grown in pots containing regular water and exposed to 16 h light/8 h dark at 26/22 °C. A total of 2-month-old seedlings with new roots and leaves were subjected to the treatment of 200 mmol/L NaCl. The leaves were harvested at 0, 6, 12, 24, 48, and 72 h for qRT-PCR with three biological replications. The samples at 0 h were used as the control. The leaves, stems, and roots were harvested at 0 and 24 h for RNA-Seq. These tissues were frozen immediately in liquid nitrogen and then stored at −80 °C.

The *Arabidopsis* seeds were treated in sodium hypochlorite solution (10% Cl, 0.05% Tween20) for 5 min, washed five times with sterilized water, and sowed on Murashige and Skoog (MS) medium (Murashige & Skoog, 1962). These Petri dishes were treated at 4 °C for 4 days and then transferred to a growth chamber under 16 h light/8 h dark cycles at 24 ± 2 °C. At the four-leaf stage, the seedlings were transferred to plastic pots containing potting mix (nutrient soil:vermiculite:perlite, 5:3:2, v/v/v). The plants were used for transformation once the secondary bolting reached a height of six to nine cm after cutting the primary bolting.

### Expression analysis of the *WRKY* genes using RNA-Seq

The leaf, stem, and root samples mentioned above were sent to the GENEWIZ Company (www.genewiz.com) for library construction and RNA-Seq (Illumina HiSeq 2500 platform). Sequencing, library construction and RNA-Seq data processing were described by Yao et al. (2016). Gene expression was quantified as fragments per kilo-base pair transcript per million mapped read (Mortazavi et al., 2008). Both the false discovery rate < 0.05 and |log_2_FC| ≥ 1 (fold change) were used to claim the DEGs. The hierarchical clustering of *WRKY* DEGs between the salt stress and the control was conducted using the Hem I 1.0 software.

### Cloning and sequence analysis of the *WRKY56* gene

The total RNA was isolated from the leaves of *P. simonii* × *P. nigra* grown under normal conditions using the Trizol reagent (Invitrogen, Carlsbad, CA, USA). The PrimeScript™ RT reagent kit with gDNA Eraser (Takara, Kusatsu, Japan) was used for first-strand cDNA synthesis. The cDNA fragment of the *WRKY56* gene was obtained by RT-PCR using a pair of primers (WRKY56-F: 5′-TCAGATCAACCCTACCCTACAC-3′ and WRKY56-R: 5′-CAGCGAGAAATTCCACACCATG-3′).

The open reading frame (ORF) of *WRKY56* was predicted using the ORF Finder (https://www.ncbi.nlm.nih.gov/orffinder/). The analysis of the physicochemical properties, such as the molecular weight (MW) and the theoretical isoelectronic point (pI) were performed with the ProtParam software (https://web.expasy.org/protparam/). The WRKY sequences of poplar and *Arabidopsis* were obtained from the plant transcription factor database (http://planttfdb.cbi.pku.edu.cn/). The phylogenetic tree was constructed using the Neighbor-Joining method in program MEGA5.0. The confidence intervals were based on 1,000 bootstrapping samples for the branches of the generated phylogenetic tree. The WRKY56 amino-acid sequence was aligned with WRKY from other plants using Clustal W2.

### Expression of the *WRKY56* gene in response to salt stress

The total RNA was extracted from the leaves of *P. simonii* × *P. nigra* under salt stress at 0, 6, 12, 24, 48, and 72 h, using a Trizol reagent (Invitrogen, Carlsbad, CA, USA). Equal amounts of RNA were used for reverse transcription to cDNA using the PrimeScript™ RT reagent kit with gDNA Eraser (Takara, Kusatsu, Japan). The cDNA served as the template in qRT-PCR using *WRKY56* gene-specific primers (WRKY56-F: 5′-TGATAATGAG GTAGAGGAGCAA-3′, WRKY56-R: 5′-AAAACCCAAAGGCGACAAGC-3′). Primers 5′-CAGCAACCGCAATACAAA-3′ and 5′-GCATACAGGGAAAGGACA-3′ were used for control amplification of *ACTIN* from poplar (EF418792). The qRT-PCR was performed on the Bio-Rad CFX96 system using the SYBR *Premix Ex Taq*™ (Takara, Kusatsu, Japan). The reaction was performed under the following conditions: initial denature at 94 °C for 30 s, 44 cycles containing denature at 94 °C for 12 s, annealing at 54 °C for 30 s, elongation at 72 °C for 40 s, and reading plate at 81 °C for 1 s.

There were three independent biological replicates at each time point. The relative expression levels of *WRKY56* were calculated using the 2^−ΔΔCt^ method, which was defined as: *C_t_* = (*C*_*t*-target_ − *C*_*t*-control_)_2_ – (*C*_*t*-target_ − *C*_*t*-control_)_1_ (Livak & Schmittgen, 2001). The analysis of variance was carried out using SPSS16.0 and Duncan’s test was used for multiple comparisons. The results were expressed as the mean ± standard deviation (SD) of three replicate experiments. The significance threshold was at *P* < 0.05.

### Vector construction and *Arabidopsis* transformation

The cDNA fragment of *WRKY56* containing ORF, with a *Bam*H I restriction site at the 5′ end and an *Xho* I site at the 3′ end was obtained by PCR using the following primers: 5′-GCGGGATCCGGTGGCGACGACTCCTGGAGCCCG-3′/5′-CGCCTC GAGCAGCGAGAAATTCCACACCATG-3′. The fragment was inserted into the *Bam*H I/*Xho* I site of the plant expression vector pBI121 to replace the *Bam*H I/*Xho* I beta-glucuronidase cassette under the control of CaMV35S promoter. The *pBI121-WRKY56* recombinant vector was transferred into *Agrobacterium EHA*105 by electroporation. *Arabidopsis* transformation was carried out by the floral dip method (Clough & Bent, 1998).

### Evaluation of transgenic *Arabidopsis* under salt stress

Transgenic *Arabidopsis* seeds from T_1_, T_2_, and T_3_ were selected on 1/2 MS medium containing 50 mg/L kanamycin under 16 h light/8 h dark cycles at 24 ± 2 °C. After selection by kanamycin resistance, the putative transgenic *Arabidopsis* lines were subjected to PCR and RT-PCR assays and the PCR fragments were further confirmed by DNA sequencing. The T_3_ homozygous seeds of the transgenic plants were selected for further growth and physiological analysis. Three transgenic lines were selected from T_3_ transgenic homozygous lines. The seeds of three T_3_ transgenic homozygous lines and a wild type (WT) were germinated in 1/2 MS medium (30 seeds/dish) supplemented with 0, 50, 100, and 150 mmol/L NaCl, with three biological replicates. The ratio of seed germination was scored after 4 days and the fresh weight (FW) was measured after 15 days. At the 4-week stage, T_3_ homozygous transgenic and wild *Arabidopsis* (as control) grown in soil were irrigated with 100 mmol/L NaCl for 5 days and the leaves were sampled for physiological trait analysis. The peroxidase (POD) and superoxide dismutase (SOD) activities were assayed as described by Mead (1976) and Giannopolitis & Ries (1997). The proline content was determined by the method described by Zhang & Huang (2013). The malondialdehyde (MDA) content was measured by the thiobarbituric acid coloration method (Hao & Liu, 2001). The results were shown as the mean ± SD of three independent experiments. Duncan’s test was used to identify significant differences between WT and transgenic *WRKY56* lines.

## Results

### Expression analysis of the WRKY genes by RNA-seq

**Figure 1:**
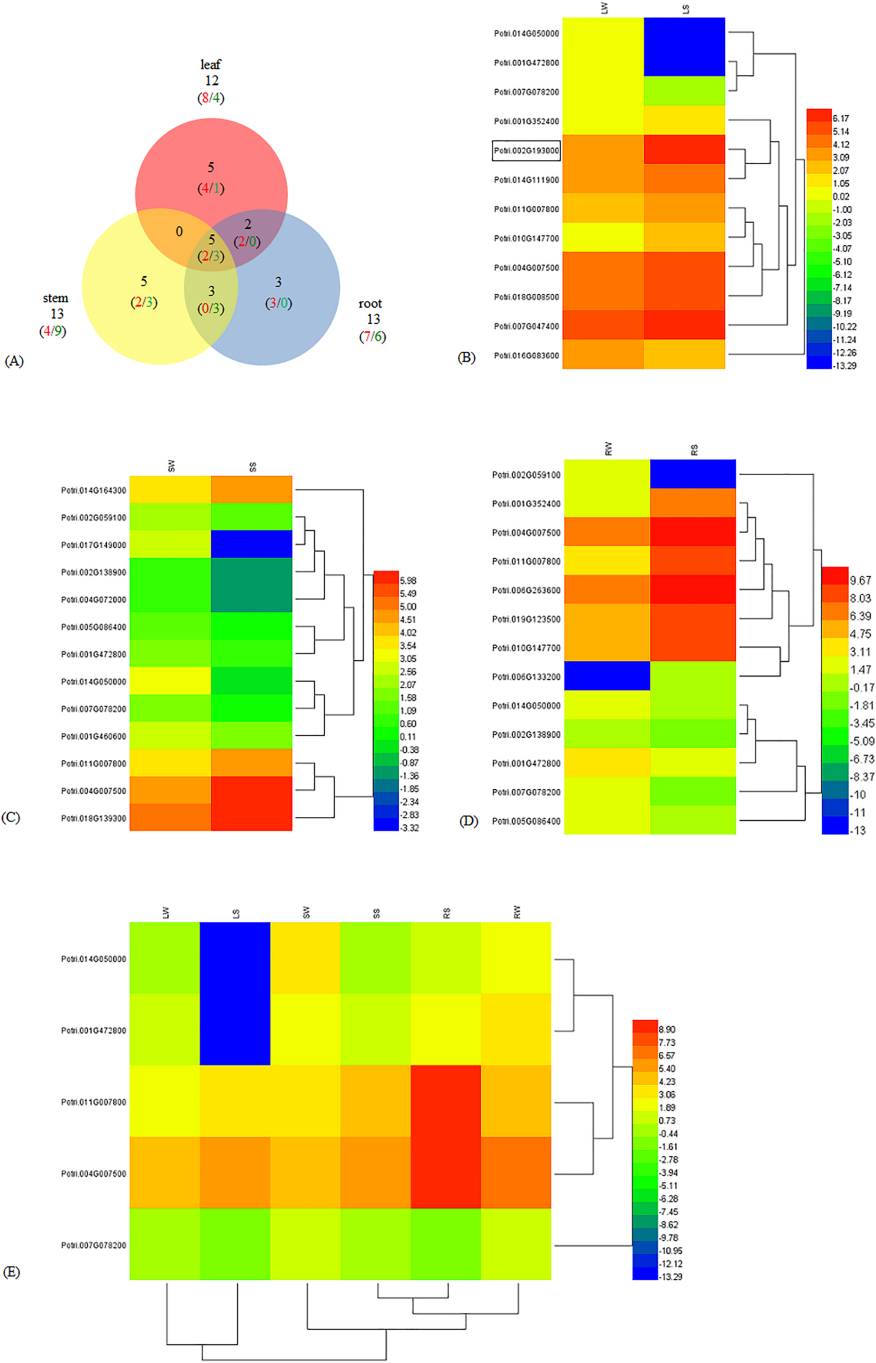
Different expression of *WRKY* genes in leaf, stem, and root of *Populus simonii* × *P. nigra* under salt stress. (A) Venn diagram of the URG and DRG in leaf, stem, and root under salt stress. The numbers in red and green showed URG and DRG, respectively. The numbers in black indicated the sum of URG and DRG. (B) Heatmap of DEGs in leaf. LW was under normal condition, and LS was treated with 200 mmol/L NaCl for 24 h. Log_2_FPKM was used to quantify the expression levels of WRKYs. Red and blue color denoted over and low expression, respectively. Potri.002G19300 with border was WRKY56. (C) Heatmap of DEGs in stem. SW was under normal condition, and SS was treated with 200 mmol/L NaCl for 24 h. (D) Heatmap of DEGs in root. RW was under normal condition, and RS was treated with 200 mmol/L NaCl for 24 h. (E) Heatmap of shared DEGs in root, stem, and leaf. LW, SW and RW were leaf, stem and root under normal condition. LS, SS, and RS were leaf, stem and root treated with 200 mmol/L NaCl for 24 h.

To understand the molecular mechanism of *WRKY* genes in response to salinity challenges, we employed RNA-Seq to obtain an overview of the transcriptome characteristics of poplar seedlings treated with NaCl and against the control. Among the 102 *WRKY* genes in poplar, we identified a total of 23 DEGs, including 13 up-regulated genes (URGs) and 10 down-regulated genes (DRGs) (Table S1). There were 12, 13, and 13 significantly DEGs in the leaf, stem, and root, respectively. Five DEGs, including two URGs and three DRGs, were shared among the leaf, stem, and root tissues. The number of DEGs shared in two out of the three tissues were zero in leaf-stem, three (zero URG/three DRGs) in stem-root, and two (two URGs/zero DRG) in leaf-root, respectively. In addition, as many as five (four URGs/one DRG), five (two URGs/three DRGs), and three (three URGs/zero DRG) DEGs were specific to the leaf, stem, and root, respectively (Fig. 1A). In the leaf, eight and four DEGs were up-regulated and down-regulated, respectively (Fig. 1B). In the stem, there were four URGs and nine DRGs (Fig. 1C). In the root, seven URGs and six DRGs were identified (Fig. 1D). The DEGs Potri.014G050000, Potri.001G472800, Potri.011G007800, Potri.004G007500 and Potri.007G078200 were shared in the leaf, stem, and root (Fig. 1E). In addition, Potri.014G050000 and Potri.001G472800, Potri.011G007800 and Potri.004G007500, were clustered together. The expression of the *WRKY56* (Potri.002G193000) gene was increased in the leaf under salt stress and that result was congruent with our previous work about qRT-PCR and cDNA-AFLP (Wang et al., 2011, 2014).

### Sequence alignments and phylogenetic analysis of the WRKY proteins

**Figure 2:**
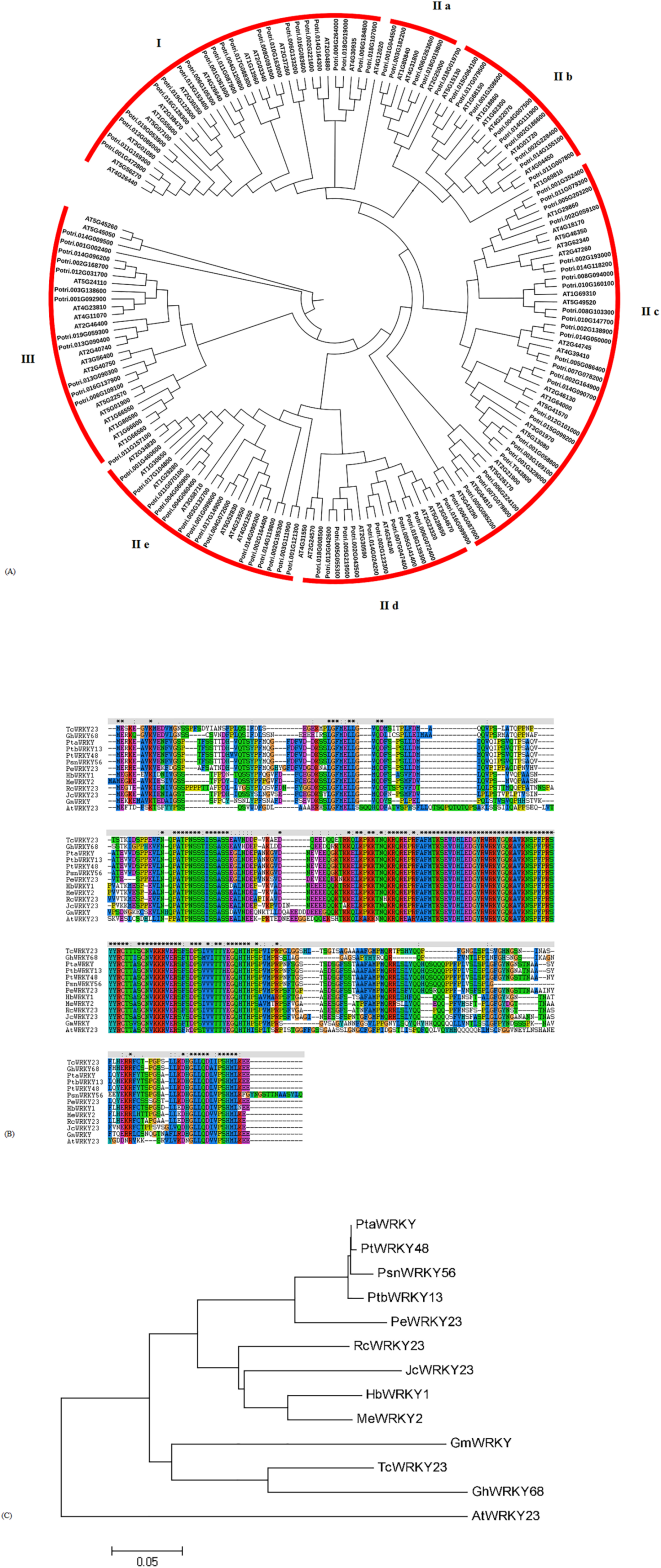
Alignment of amino-acid sequence and phylogenetic tree analysis of WRKYs from poplar and other plants. (A) Phylogenetic tress of all WRKY proteins from poplar and Arabidopsis. (B) Amino-acid sequences alignment of WRKYs from different plant species by Clustal W2. (C) Phylogenetic tree analysis of WRKYs from different plant species by neighbor-joining method. GenBank accession numbers of all amino acid sequences were as follows: PtaWRKY (*Populus tremula* × *Populus alba*) (ABK41486), PtbWRKY13 ((*Populus tomentosa* × *Populus bolleana*) × *Populus tomentosa*) (ACV92015), PeWRKY23 (*Populus euphratica*) (XP_011040950), PtWRKY48 (*Populus trichocarpa*) (XP_002301524), HbWRKY1 (*Hevea brasiliensis*) (ADF45433), MeWRKY2 (*Manihot esculenta*) (AMO00370), RcWRKY23 (*Ricinus communis*) (XP_002515251), JcWRKY23 (*Jatropha curcas*) (XP_012082928), TcWRKY23 (*Theobroma cacao*) (XP_017983061), GhWRKY68 (*Gossypium hirsutum*) (AIS23529), GmWRKY (*Glycine max*) (AJB84597), AtWRKY23 (*Arabidopsis thaliana*) (AT2G47260).

To investigate the evolutionary relationship of WRKYs in poplar and *Arabidopsis*, 102 WRKYs from poplar and 72 WRKYs from *Arabidopsis* were downloaded to construct a phylogenetic tree. Based on the results from the sequence alignments and the phylogenetic tree, the WRKYs were classified into three groups: group I, group II, and group III (Fig. 2A). In poplar, 22 WRKYs containing two WRKY domains and a C_2_H_2_ zinc finger motif were assigned to group I. 68 WRKYs with one WRKY domain and a C_2_H_2_ zinc finger motif were categorized into group II, which was further classified into five subgroups, designated as group II-a (5), group II-b (9), group II-c (27), group II-d (11), and group II-e (16). 12 WRKYs, each harboring one WRKY domain and a C_2_CH zinc finger motif, were identified to group III. According to the phylogenetic tree, we found that the WRKY56 (Potri.002G193000) belonged to group II-c.

### Cloning and sequence analysis of the *WRKY56* gene

The full-length cDNA of the *WRKY56* gene was isolated from the leaves of *P. simonii* × *P. nigra* by RT-PCR, and was 1,177 bp and contained an ORF of 954 bp encoding 317 amino-acid residues. The MW of the WRKY56 was 35.37 kDa with theoretical pI of 6.67. The conserved domain included the WRKY domain with 60 amino acids. Evidence from the sequence alignment revealed that all of the 13 WRKY proteins shared one WRKY domain and C_2_H_2_ zinc finger motif (Fig. 2B). The WRKY56 from *P. simonii* × *P. nigra* showed a high homology with the WRKY proteins from other poplars, but a longer evolutionary distance with the WRKY proteins from *Gossypium hirsutum*, *Theobroma cacao*, *Glycine max*, and *A. thaliana* (Fig. 2C).

### Expression characterization of the *WRKY56* gene under salt stress by qRT-PCR

**Figure 3:**
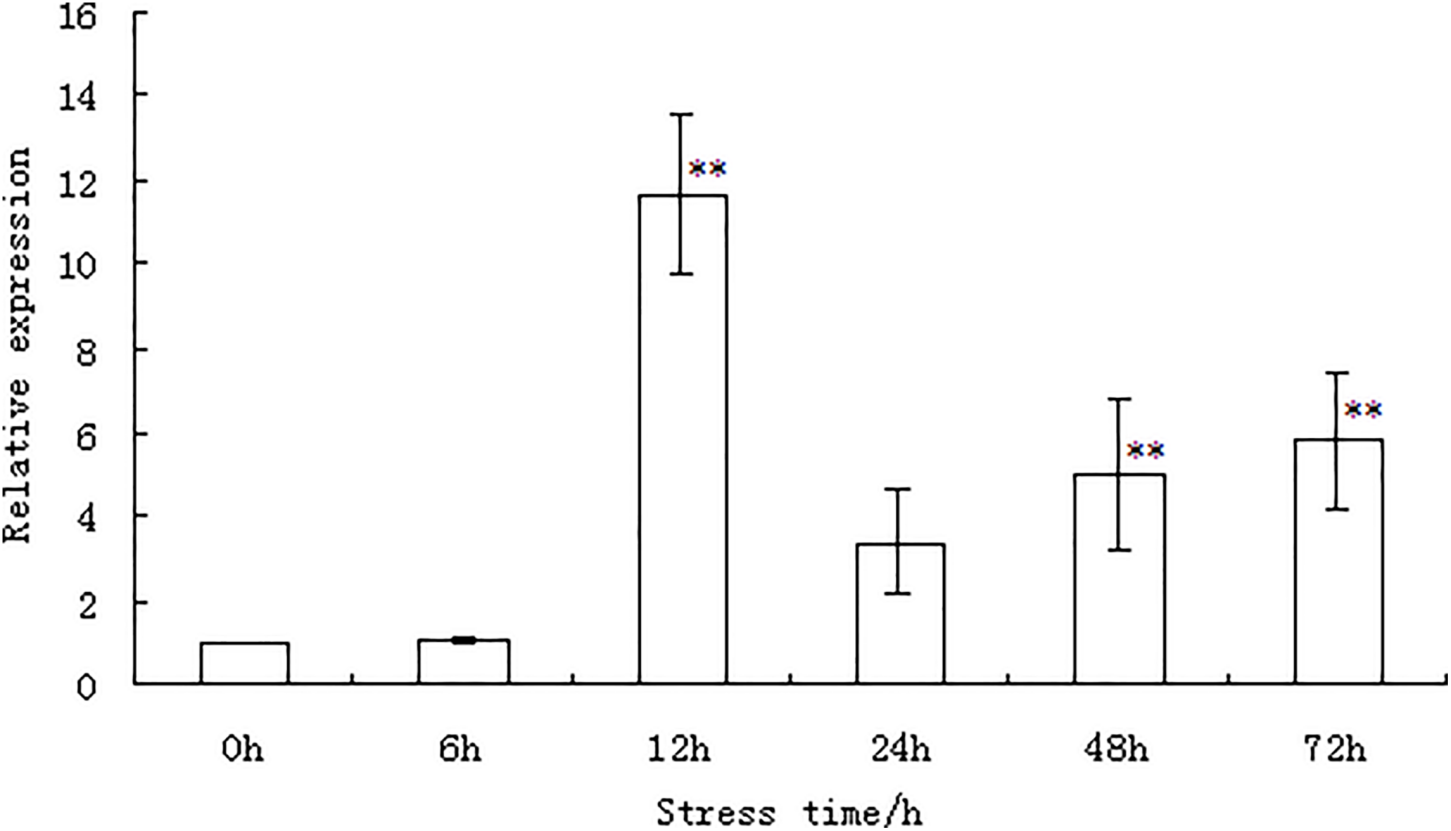
*WRKY56* gene expression in leaves of *Populus simonii* × *P. nigra* under salt stress. *Actin* gene from *Populus simonii* × *P. nigra* was the reference gene. The results were expressed as the mean ± standard deviation (SD) of three replicate experiments. ** indicates significant difference at *P* < 0.01 level.

The expression of the *WRKY56* gene in the leaves of *P. simonii* × *P. nigra* under salt stress was examined by qRT-PCR at different time points as shown in Fig. 3 and Table S2. Under salt stress, the expression of the *WRKY56* gene initially increased between 6–12 h. The peak value appeared at 12 h and then decreased between 12 and 72 h. Evidence from statistical analysis indicated that the expression of the *WRKY56* gene was significantly higher than that of the control at 12, 48, and 72 h (*P* < 0.01), respectively.

### Characterization of transgenic *Arabidopsis* over-expressing the *WRKY56* gene under salt stress

**Figure 4:**
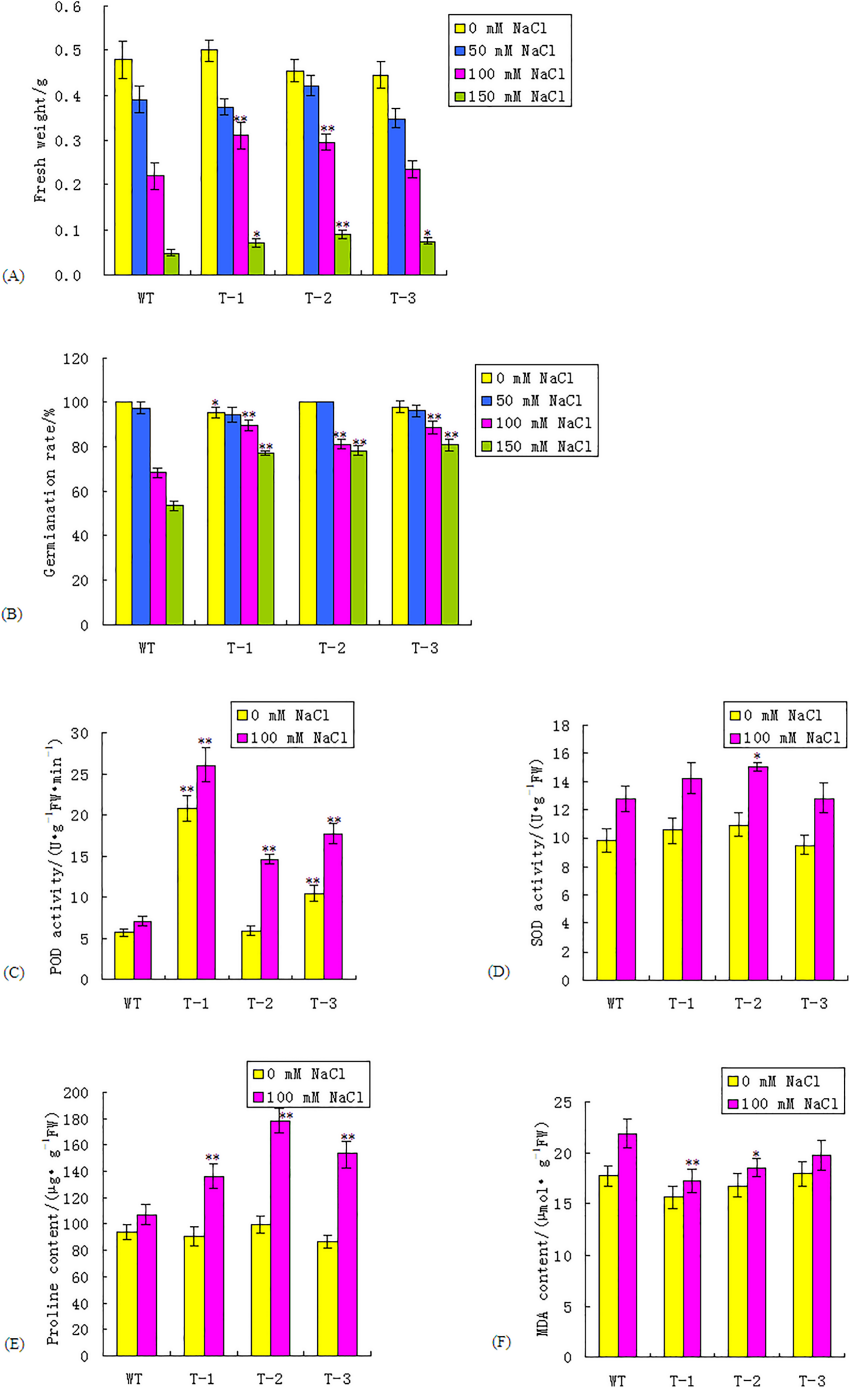
Comparisons of fresh weight, seed germination rate, POD and SOD activity, proline and MDA content between WT and transgenic lines under normal and salt stress condition. (A–F) Fresh weight, seed germination rate, POD activity, SOD activity, proline and MDA content respectively between WT and transgenic lines. WT, wild type. T-1, T-2, T-3, transgenic *WRKY56* lines. Mean values and standard deviations were calculated from three independent experiments. * and ** indicate significant difference at *P* < 0.05 and *P* < 0.01 level.

To characterize the growth of transgenic *Arabidopsis* under salt stress, the three transgenic homozygous lines from the T_3_ generation and the WT were sown into 1/2 MS medium supplemented with 0, 50, 100, and 150 mmol/L NaCl. The FWs were measured after 15 days. The results revealed that there was no significant difference in FW between the transgenic lines and the WT under both normal and 50 mmol/L NaCl conditions. The FWs of the transgenic lines and the WT were decreased under salt stress, compared with those under normal conditions. Transgenic lines exhibited a greater FW than that of the WT under 100 and 150 mmol/L NaCl conditions (Fig. 4A; Table S3). The FWs of the transgenic lines T-1 and T-2, under 100 mmol/L NaCl conditions, were 40.9% and 36.36% higher, respectively, than those of the WT, which was significant (*P* < 0.01). The FWs of the transgenic lines were higher than those of the WT plants under the 150 mmol/L NaCl treatment; the FW of T-2 was 80% higher than the WT plants, which was significant (*P* < 0.01).

To evaluate the impact of the *WRKY56* gene on seed germination, WT and three transgenic homozygous lines seeds from the T_3_ generation were germinated in 1/2 MS medium supplemented with 0, 50, 100, and 150 mmol/L NaCl, respectively. The seed germination rates of the WT and the three transgenic lines were similar to the trends found in the FW. There was no extremely significant difference in the germination rate between the transgenic lines and the WT under normal and 50 mmol/L NaCl conditions. Under both 100 and 150 mmol/L NaCl conditions, transgenic seeds exhibited a higher germination rate than those of the WT. With the challenge of 100 and 150 mmol/L NaCl, the seed germination rates of the transgenic lines were 18.97–31.17% and 44.71–51.62% higher than those of the WT plants, respectively (*P* < 0.01) (Fig. 4B; Table S3). These results indicated that the *WRKY56* gene could enhance the salt tolerance of *Arabidopsis*.

The SOD and POD activities of the transgenic *WRKY56* lines were assayed under normal and 100 mmol/L NaCl conditions. Under normal conditions, the POD activities of T-1 and T-3 were 3.61 and 1.82 times higher than those of the WT, which was significant at *P* < 0.01. Under 100 mmol/L NaCl stress, the POD activities of the transgenic lines were 2.08–3.70 times higher than those of the WT plants, which was significant at *P* < 0.01 (Fig. 4C; Table S4). The SOD and POD activities of the transgenic lines and the WT under both normal and NaCl conditions showed different trends. Under the normal and 100 mmol/L NaCl conditions, the difference of SOD activity between the transgenic lines and the WT was not significant except that the SOD activity of T-2 was 17.07% higher than that of WT under the 100 mmol/L NaCl conditions, which was significant at *P* < 0.05 (Fig. 4D; Table S4).

The proline content of the WT was similar to that of the transgenic *WRKY56* lines under the normal condition; however, the proline content of the transgenic lines was 27.10–66.41% higher than that of the WT under 100 mmol/L NaCl conditions, with a significance of *P* < 0.01 (Fig. 4E; Table S4). These results demonstrated that an over-expression of the *WRKY56* gene in *Arabidopsis* under salt stress could elevate the SOD and POD activities, as well as proline content in transgenic plants.

There was no extremely significant difference in the MDA contents between the transgenic lines and the WT under normal conditions. The MDA contents between the WT and transgenic lines under salt stress were higher than those under normal conditions. The MDA contents of the transgenic lines under salt stress were 9.93–21.05% lower than those of the WT plants, especially T-1, with a significance of *P* < 0.01 (Fig. 4F; Table S3). These results indicated that an over-expression of the *WRKY56* gene in *Arabidopsis* could reduce the lipid over-oxidation rate in transgenic plants.

## Discussion

As a valuable forest resource and important ecology species, poplar is the model plant for studies on forest physiology and genetic engineering. With the completion of the genome sequencing of poplar, it has provided a genetic basis for fully understanding the molecular mechanism of the stress response. Genome-wide analysis of poplar has identified 2,576 TFs which can be classified into 64 different families including that of the WRKY (Rushton et al., 2010). In this study, we identified 23 DEGs from 102 *WRKY* genes, including 13 URGs and 10 DRGs. Five DEGs were shared among the leaf, stem, and root tissues. Potri.014G050000 and Potri.001G472800, and Potri.011G007800 and Potri.004G007500 from these five DEGs were clustered together, indicating that the DEGs might share similar regulatory mechanisms under salt stress. We also found that 18 of the 23 *WRKY* DEGs were expressed in a tissue-specific manner when subject to salt stress. The results indicated that these genes might play important but diverse roles in different tissues in response to salinity. Evidence from genome-wide gene expression profiling of the WRKY family in other plants also indicated that the *WRKY* genes could be induced by abiotic stress, such as salt (Diao et al., 2016; Song et al., 2016), heat shock (Wu et al., 2016), and drought (Huang et al., 2015). In this study, evidence from RNA-Seq and qRT-PCR demonstrated that the expression of the *WRKY56* gene (locus name: Potri.002G193000) was significantly induced by salt stress. These results were consistent with our previous studies which indicated that 13 out of 119 poplar *WRKY* genes including *WRKY56*, showed temporal and spatial expression patterns under salt stress (Wang et al., 2014). In our earlier work, the cDNA-AFLP method was also used to screen the different expressing genes of *P. simonii* × *P. nigra* in response to salt stress. We found that the expression of the *WRKY56* gene was induced by salt stress (Wang et al., 2011). In summary, the results demonstrated that the *WRKY56* gene was responsive to salt stress and expression of the *WRKY56* was up-regulated under salt stress. However, the function of *WRKY56* has been unknown, especially under salt stress.

*Arabidopsis thaliana* is a model plant in the field of plant physiology and molecular biology due to the advantages of a small genome, short growth cycle, quantity of seeds, self-pollination, easy transformation, and phenotypic observation (Meinke et al., 1998). There are many reports about the heterologous expression of woody plant genes in *A. thaliana* in order to investigate the gene functions. For example, the overexpression of PeSCL7 from *P. euphratica* by transgenic *Arabidopsis* revealed an enhanced tolerance to drought and salt treatments. The activities of amylase and SOD were increased in transgenic *Arabidopsis* (Ma et al., 2010). In our study, the over-expression of the *WRKY56* gene by transgenic *Arabidopsis* showed a significant increase in the germination rate, FW, proline content, and SOD and POD activities, but a decrease of MDA accumulation when compared to the WT. These results indicated that the expression of the exgenous *WRKY56* gene increased the ability of reactive oxygen species (ROS) to clean up, giving rise to a stronger tolerance to salt stress, compared to the WT. Under salt stress, plant cells generated a large number of ROS, including H_2_O_2_, ·OH, ^1^O_2_, O_2_·^−^, RO·, ROO·. The SOD was the first key enzyme to scavenge the ROS in reactive oxygen metabolism due to its ability to transform O_2_^−^ into H_2_O_2_ in a disproportionation reaction. The H_2_O_2_ could be cleared up through the GSH-AsA cycle or through degradation by catalase and POD. Ultimately, H_2_O_2_ was decomposed into H_2_O and O_2_ (Alscher, Erturk & Heath, 2002). The SOD and POD enzymes have an important role in protecting cells against salt stress. Plants could protect their cells under stress conditions by increasing an antioxidant enzyme, such as SOD and POD, and by increasing protective substances, such as proline, which is a major organic osmolyte in stabilizing sub-cellular structures and scavenging free radicals under stress conditions (Ashraf & Foolad, 2007). Proline accumulation was considered to influence stress tolerance of the plant in redox balance, osmoprotection, signaling, and translation (Szabados & Savoure, 2010). The MDA was a decomposition product of polyunsaturated fatty acids and its accumulation indicates an increasing production of a superoxide radical, hydrogen peroxide, in plants under stress conditions (Xie et al., 2008). Our studies demonstrated that *WRKY56* might play a positive role in a plant’s response to salt stress and over-expression of the *WRKY56* gene can enhance ROS scavenging capacity to improve the salt tolerance of transgenic *Arabidopsis*. Further research will help to define whether the overexpression of WRKY56 can enhance the salt tolerance of the transgenic poplar.

## Conclusion

In conclusion, we identified 23 *WRKY* genes in which the expression varied significantly in a tissue-specific manner in response to salt stress in *P. simonii* × *P. nigra*. The expression of the *WRKY56* gene was increased under salt stress. The *WRKY56* gene could improve the tolerance of transgenic *Arabidopsis* to salt stress. These results provide a theoretical basis for functional research on the *WRKY* genes and indicate that the *WRKY56* gene may potentially find an application as a genetic resource in genetic engineering breeding.

## Supplemental Information

10.7717/peerj.7291/supp-1 Supplemental Information 1 The DEGs of *WRKY* in leaf, stem and root of *Populus simonii* × *P. nigra* under salt stress. A total of 23 different expressed *WRKYs* in leaf, stem and root of *Populus simonii* × *P. nigra* under 200 mmol/L NaCl for 24 h were obtained by RNA-Seq.

10.7717/peerj.7291/supp-2 Supplemental Information 2 Expression levels of *WRKY56* gene in leaves of *Populus simonii* × *P. nigra* under salt stress. Values are Mean ± SD (*n* = 3). Upper- and lowercase letters indicate significant difference at *P* < 0.01 and *P* < 0.05 using Duncant test, respectively.

10.7717/peerj.7291/supp-3 Supplemental Information 3 Comparisons of fresh weight and seed germination rate between WT and transgenic lines under normal and salt stress condition. Mean values and deviations were calculated from three independent experiments. WT: wild type. T-1, T-2, T-3: transgenic *WRKY56* lines. Upper- and lowercase letters mean at *P* < 0.01 and *P* < 0.05 significant level.

10.7717/peerj.7291/supp-4 Supplemental Information 4 Comparisons of POD activity, SOD activity, proline and MDA content between WT and transgenic lines under normal and salt stress condition. Values are mean ± SD based on three replicates. WT: wild type. T-1 to T-3: transgenic *WRKY56* lines. Uppercase letters were significant difference at *P* < 0.01 and lowercase letters were significant difference at *P* < 0.05.

10.7717/peerj.7291/supp-5 Supplemental Information 5 Expression level of WRKY56 under salt stress. The raw data of real-time PCR were applied for data analysis and preparation of Fig. 3 and Table S2.

10.7717/peerj.7291/supp-6 Supplemental Information 6 Fresh weight, seed germination rate, POD activity, SOD activity, proline and MDA content between WT and transgenic lines under normal and salt stress conditions. The raw data of growth and physiological traits were applied for data analysis and preparation of Fig. 4 and Tables S3–S4.

## Additional Information and Declarations

### Data Availability

The following information was supplied regarding data availability: The raw measurements are available in the Tables S1–S4 and Data S1–S2.
