# Supplementary material for: Association of transcription factor WRKY56 gene from Populus simonii × P. nigra with salt tolerance in Arabidopsis thaliana
Source: PeerJ. 2019 Jul 9;7:e7291. doi: 10.7717/peerj.7291 (PMC6625503; doi:10.7717/peerj.7291)
Supplement: Supplemental Information 2 — Values are Mean ± SD (n = 3). Upper- and lowercase letters indicate significant difference at P < 0.01 and P < 0.05 using Duncant test, respectively. [file peerj-07-7291-s002.doc]

**Supplementary Table 2** Expression levels of WRKY56 gene in leaves of Populus simonii×P. nigra under salt stress.

| Time | Expression level of *WRKY56* under salt stress |
| --- | --- |
| 0 h | 1±0 aA |
| 6 h | 1.08±0.08 aA |
| 12 h | 11.64±1.90 bB |
| 24 h | 3.37±1.28 acAC |
| 48 h | 5.02±1.83 cC |
| 72 h | 5.8±1.60 cC |

Note: Values are Mean ± SD (n=3). Upper- and lowercase letters indicate significant difference at *P<0.01* and *P<0.05* using Duncant test, respectively.
