# Supplementary material for: Association of transcription factor WRKY56 gene from Populus simonii × P. nigra with salt tolerance in Arabidopsis thaliana
Source: PeerJ. 2019 Jul 9;7:e7291. doi: 10.7717/peerj.7291 (PMC6625503; doi:10.7717/peerj.7291)
Supplement: Supplemental Information 3 — Mean values and deviations were calculated from three independent experiments. WT: wild type. T-1, T-2, T-3: transgenic WRKY56 lines. Upper- and lowercase letters mean at P < 0.01 and P < 0.05 significant level. [file peerj-07-7291-s003.doc]

**Supplementary Table 3** Comparisons of fresh weight and seed germination rate between WT and transgenic lines under normal and salt stress condition.

|  | Fresh weight /g | | | | Seed germination rate /% | | | |
| --- | --- | --- | --- | --- | --- | --- | --- | --- |
|  | 0 mM NaCl | 50 mM NaCl | 100mM NaCl | 150 mM NaCl | 0 mM NaCl | 50 mM NaCl | 100mM NaCl | 150mM NaCl |
| WT | 0.48±0.04 a | 0.39±0.03abA | 0.22±0.03aA | 0.05±0.01aA | 100±0aA | 97.31±2.14abA | 68.33±1.92aA | 53.43±1.84aA |
| T-1 | 0.50±0.03 a | 0.38±0.02abA | 0.31±0.03 bB | 0.07±0.01bAB | 95.45±2.45bA | 94.18±3.32bA | 89.63±2.38bB | 77.32±1.18bB |
| T-2 | 0.46±0.03a | 0.42±0.02 bA | 0.30±0.02 bBC | 0.09±0.01bB | 100±0aA | 100±0aA | 81.29±2.35cC | 78.24±2.36bB |
| T-3 | 0.45±0.03a | 0.35±0.02 aA | 0.24±0.02 aAC | 0.08±0.01 bAB | 97.87±2.67abA | 96.32±2.42abA | 88.40±2.87 bB | 81.01±2.53bB |

Note: Values are Mean ± SD (n=3). Upper- and lowercase letters indicate significant difference at *P<0.01* and *P<0.05* using Duncant test, respectively.
