# Supplementary material for: Association of transcription factor WRKY56 gene from Populus simonii × P. nigra with salt tolerance in Arabidopsis thaliana
Source: PeerJ. 2019 Jul 9;7:e7291. doi: 10.7717/peerj.7291 (PMC6625503; doi:10.7717/peerj.7291)
Supplement: Supplemental Information 4 — Values are mean ± SD based on three replicates. WT: wild type. T-1 to T-3: transgenic WRKY56 lines. Uppercase letters were significant difference at P < 0.01 and lowercase letters were significant difference at P < 0.05. [file peerj-07-7291-s004.doc]

**Supplementary Table 4** Comparisons of POD activity, SOD activity, proline and MDA content between WT and transgenic lines under normal and salt stress condition.

|  | POD activity/(U•g^-1^FW•min^-1^) | | SOD activity/(U•g^-1^FW) | | Proline content/(µg• g^-1^FW) | | MDA content/(µmol• g^-1^FW) | |
| --- | --- | --- | --- | --- | --- | --- | --- | --- |
|  | 0 mM NaCl | 100 mM NaCl | 0 mM NaCl | 100 mM NaCl | 0 mM NaCl | 100 mM NaCl | 0 mM NaCl | 100 mM NaCl |
| WT | 5.74±0.47 aA | 7.04±0.60 aA | 9.84±0.83a | 12.83±0.89 aA | 93.78±5.59 a | 107.34±7.46 aA | 17.79±1.00 abA | 21.95±1.45 aA |
| T-1 | 20.73±1.55 bB | 26.06±2.02 bB | 10.54±0.96 a | 14.23±1.04 abA | 90.34±7.32 a | 136.43±9.12 bB | 15.65±1.15 aA | 17.33±1.10 bB |
| T-2 | 5.87±0.54 aA | 14.61±0.65 cC | 10.95±0.86 a | 15.02±0.25 bA | 99.26±6.54 a | 178.62±9.91 cC | 16.81±1.17 abA | 18.58±0.93 bAB |
| T-3 | 10.47±0.95 cC | 17.67±1.20 dC | 9.53±0.70 a | 12.85±1.07 aA | 86.96±4.96 a | 153.38±9.94 bB | 17.99±1.16 bA | 19.77±1.45 abAB |

Note: Values are Mean ± SD (n=3). Upper- and lowercase letters indicate significant difference at *P<0.01* and *P<0.05* using Duncant test, respectively.
